# Climate sensitivity, sea level and atmospheric carbon dioxide

**DOI:** 10.1098/rsta.2012.0294

**Published:** 2013-10-28

**Authors:** James Hansen, Makiko Sato, Gary Russell, Pushker Kharecha

**Affiliations:** 1 The Earth Institute, Columbia University, New York, NY 10027, USA; 2 NASA Goddard Institute for Space Studies, New York, NY 10027, USA

**Keywords:** climate, climate sensitivity, palaeoclimate, sea level

## Abstract

Cenozoic temperature, sea level and CO_2_ covariations provide insights into climate sensitivity to external forcings and sea-level sensitivity to climate change. Climate sensitivity depends on the initial climate state, but potentially can be accurately inferred from precise palaeoclimate data. Pleistocene climate oscillations yield a fast-feedback climate sensitivity of 3±1^°^C for a 4 W m^−2^ CO_2_ forcing if Holocene warming relative to the Last Glacial Maximum (LGM) is used as calibration, but the error (uncertainty) is substantial and partly subjective because of poorly defined LGM global temperature and possible human influences in the Holocene. Glacial-to-interglacial climate change leading to the prior (Eemian) interglacial is less ambiguous and implies a sensitivity in the upper part of the above range, i.e. 3–4^°^C for a 4 W m^−2^ CO_2_ forcing. Slow feedbacks, especially change of ice sheet size and atmospheric CO_2_, amplify the total Earth system sensitivity by an amount that depends on the time scale considered. Ice sheet response time is poorly defined, but we show that the slow response and hysteresis in prevailing ice sheet models are exaggerated. We use a global model, simplified to essential processes, to investigate state dependence of climate sensitivity, finding an increased sensitivity towards warmer climates, as low cloud cover is diminished and increased water vapour elevates the tropopause. Burning all fossil fuels, we conclude, would make most of the planet uninhabitable by humans, thus calling into question strategies that emphasize adaptation to climate change.

## 1. Introduction

Humanity is now the dominant force driving changes in the Earth's atmospheric composition and climate [1]. The largest climate forcing today, i.e. the greatest imposed perturbation of the planet's energy balance [1,2], is the human-made increase in atmospheric greenhouse gases (GHGs), especially CO_2_ from the burning of fossil fuels.

Earth's response to climate forcings is slowed by the inertia of the global ocean and the great ice sheets on Greenland and Antarctica, which require centuries, millennia or longer to approach their full response to a climate forcing. This long response time makes the task of avoiding dangerous human alteration of climate particularly difficult, because the human-made climate forcing is being imposed rapidly, with most of the current forcing having been added in just the past several decades. Thus, observed climate changes are only a partial response to the current climate forcing, with further response still ‘in the pipeline’ [3].

Climate models, numerical climate simulations, provide one way to estimate the climate response to forcings, but it is difficult to include realistically all real-world processes. Earth's palaeoclimate history allows empirical assessment of climate sensitivity, but the data have large uncertainties. These approaches are usually not fully independent, and the most realistic eventual assessments will be ones combining their greatest strengths.

We use the rich climate history of the Cenozoic era in the oxygen isotope record of ocean sediments to explore the relation of climate change with sea level and atmospheric CO_2_, inferring climate sensitivity empirically. We use isotope data from Zachos *et al.* [4], which are improved over data used in our earlier study [5], and we improve our prescription for separating the effects of deep ocean temperature and ice volume in the oxygen isotope record as well as our prescription for relating deep ocean temperature to surface air temperature. Finally, we use an efficient climate model to expand our estimated climate sensitivities beyond the Cenozoic climate range to snowball Earth and runaway greenhouse conditions.

## 2. Overview of Cenozoic climate and our analysis approach

**Figure 1.**
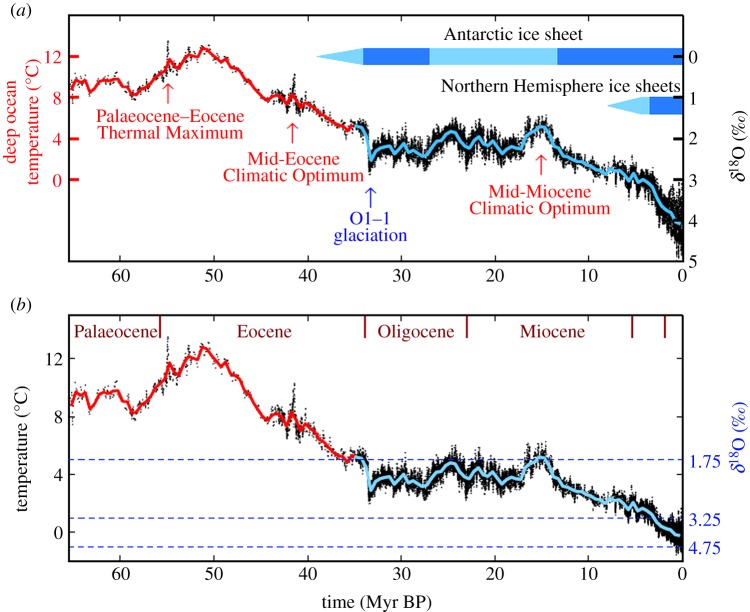
(*a*) Global deep ocean *δ*^18^O from Zachos *et al.* [4] and (*b*) estimated deep ocean temperature based on the prescription in our present paper. Black data points are five-point running means of the original temporal resolution; red and blue curves have a 500 kyr resolution. Coarse temporal sampling reduces the amplitude of glacial–interglacial oscillations in the intervals 7–17, 35–42 and 44–65 Myr BP.

The Cenozoic era, the past 65.5 million years (Myr), provides a valuable perspective on climate [5,6] and sea-level change [7], and Cenozoic data help clarify our analysis approach. The principal dataset we use is the temporal variation of the oxygen isotope ratio (*δ*^18^O relative to *δ*^16^O; figure 1*a* right-hand scale) in the shells of deep-ocean-dwelling microscopic shelled animals (foraminifera) in a near-global compilation of ocean sediment cores [4]. *δ*^18^O yields an estimate of the deep ocean temperature (figure 1*b*), as discussed in §3. Note that coarse temporal resolution of *δ*^18^O data in the intervals 7–17, 35–42 and 44–65 Myr reduces the apparent amplitude of glacial–interglacial climate fluctuations (see electronic supplementary material, figure S1). We use additional proxy measures of climate change to supplement the *δ*^18^O data in our quantitative analyses.

Carbon dioxide is involved in climate change throughout the Cenozoic era, both as a climate forcing and as a climate feedback. Long-term Cenozoic temperature trends, the warming up to about 50 Myr before present (BP) and subsequent long-term cooling, are likely to be, at least in large part, a result of the changing natural source of atmospheric CO_2_, which is volcanic emissions that occur mainly at continental margins due to plate tectonics (popularly ‘continental drift’); tectonic activity also affects the weathering sink for CO_2_ by exposing fresh rock. The CO_2_ tectonic source grew from 60 to 50 Myr BP as India subducted carbonate-rich ocean crust while moving through the present Indian Ocean prior to its collision with Asia about 50 Myr BP [8], causing atmospheric CO_2_ to reach levels of the order of 1000 ppm at 50 Myr BP [9]. Since then, atmospheric CO_2_ declined as the Indian and Atlantic Oceans have been major depocentres for carbonate and organic sediments while subduction of carbonate-rich crust has been limited mainly to small regions near Indonesia and Central America [10], thus allowing CO_2_ to decline to levels as low as 170 ppm during recent glacial periods [11]. A climate forcing due to a CO_2_ change from 1000 to 170 ppm is more than 10 W m^−2^, which compares with forcings of the order of 1 W m^−2^ for competing climate forcings during the Cenozoic era [5], specifically long-term change of solar irradiance and change of planetary albedo (reflectance) owing to the overall minor displacement of continents in that era.

Superimposed on the long-term trends are occasional global warming spikes, ‘hyperthermals’, most prominently the Palaeocene–Eocene Thermal Maximum (PETM) at approximately 56 Myr BP [12] and the Mid-Eocene Climatic Optimum at approximately 42 Myr BP [13], coincident with large temporary increases of atmospheric CO_2_. The most studied hyperthermal, the PETM, caused global warming of at least 5^°^C coincident with injection of a likely 4000–7000 Gt of isotopically light carbon into the atmosphere and ocean [14]. The size of the carbon injection is estimated from changes in the stable carbon isotope ratio ^13^C/^12^C in sediments and from ocean acidification implied by changes in the ocean depth below which carbonate dissolution occurred.

The potential carbon source for hyperthermal warming that received most initial attention was methane hydrates on continental shelves, which could be destabilized by sea floor warming [15]. Alternative sources include release of carbon from Antarctic permafrost and peat [16]. Regardless of the carbon source(s), it has been shown that the hyperthermals were astronomically paced, spurred by coincident maxima in the Earth's orbit eccentricity and spin axis tilt [17], which increased high-latitude insolation and warming. The PETM was followed by successively weaker astronomically paced hyperthermals, suggesting that the carbon source(s) partially recharged in the interim [18]. A high temporal resolution sediment core from the New Jersey continental shelf [19] reveals that PETM warming in at least that region began about 3000 years prior to a massive release of isotopically light carbon. This lag and climate simulations [20] that produce large warming at intermediate ocean depths in response to initial surface warming are consistent with the concept of a methane hydrate role in hyperthermal events.

The hyperthermals confirm understanding about the long recovery time of the Earth's carbon cycle [21] and reveal the potential for threshold or ‘tipping point’ behaviour with large amplifying climate feedback in response to warming [22]. One implication is that if humans burn most of the fossil fuels, thus injecting into the atmosphere an amount of CO_2_ at least comparable to that injected during the PETM, the CO_2_ would stay in the surface carbon reservoirs (atmosphere, ocean, soil, biosphere) for tens of thousands of years, long enough for the atmosphere, ocean and ice sheets to fully respond to the changed atmospheric composition. In addition, there is the potential that global warming from fossil fuel CO_2_ could spur release of CH_4_ and CO_2_ from methane hydrates or permafrost. Carbon release during the hyperthermals required several thousand years, but that long injection time may have been a function of the pace of the astronomical forcing, which is much slower than the pace of fossil fuel burning.

The Cenozoic record also reveals the amplification of climate change that occurs with growth or decay of ice sheets, as is apparent at about 34 Myr BP when the Earth became cool enough for large-scale glaciation of Antarctica and in the most recent 3–5 Myr with the growth of Northern Hemisphere ice sheets. Global climate fluctuated in the 20 Myr following Antarctic glaciation with warmth during the Mid-Miocene Climatic Optimum (MMCO, 15 Myr BP) possibly comparable to that at 34 Myr BP, as, for example, Germany became warm enough to harbour snakes and crocodiles that require an annual temperature of about 20^°^C or higher and a winter temperature more than 10^°^C [23]. Antarctic vegetation in the MMCO implies a summer temperature of approximately 11^°^C warmer than today [24] and annual sea surface temperatures ranging from 0^°^C to 11.5^°^C [25].

Superimposed on the long-term trends, in addition to occasional hyperthermals, are continual high-frequency temperature oscillations, which are apparent in figure 1 after 34 Myr BP, when the Earth became cold enough for a large ice sheet to form on Antarctica, and are still more prominent during ice sheet growth in the Northern Hemisphere. These climate oscillations have dominant periodicities, ranging from about 20 to 400 kyr, that coincide with variations in the Earth's orbital elements [26], specifically the tilt of the Earth's spin axis, the eccentricity of the orbit and the time of year when the Earth is closest to the Sun. The slowly changing orbit and tilt of the spin axis affect the seasonal distribution of insolation [27], and thus the growth and decay of ice sheets, as proposed by Milankovitch [28]. Atmospheric CO_2_, CH_4_ and N_2_O have varied almost synchronously with global temperature during the past 800 000 years for which precise data are available from ice cores, the GHGs providing an amplifying feedback that magnifies the climate change instigated by orbit perturbations [29–31].

Ocean and atmosphere dynamical effects have been suggested as possible causes of some climate change within the Cenozoic era; for example, topographical effects of mountain building [32], closing of the Panama Seaway [33] or opening of the Drake Passage [34]. Climate modelling studies with orographic changes confirm significant effects on monsoons and on Eurasian temperature [35]. Modelling studies indicate that closing of the Panama Seaway results in a more intense Atlantic thermohaline circulation, but only small effects on Northern Hemisphere ice sheets [36]. Opening of the Drake Passage surely affected ocean circulation around Antarctica, but efforts to find a significant effect on global temperature have relied on speculation about possible effects on atmospheric CO_2_ [37]. Overall, there is no strong evidence that dynamical effects are a major direct contributor to Cenozoic global temperature change.

We hypothesize that the global climate variations of the Cenozoic (figure 1) can be understood and analysed via slow temporal changes in Earth's energy balance, which is a function of solar irradiance, atmospheric composition (specifically long-lived GHGs) and planetary surface albedo. Using measured amounts of GHGs during the past 800 000 years of glacial–interglacial climate oscillations and surface albedo inferred from sea-level data, we show that a single empirical ‘fast-feedback’ climate sensitivity can account well for the global temperature change over that range of climate states. It is certain that over a large climate range climate sensitivity must become a strong function of the climate state, and thus we use a simplified climate model to investigate the dependence of climate sensitivity on the climate state. Finally, we use our estimated state-dependent climate sensitivity to infer Cenozoic CO_2_ change and compare this with proxy CO_2_ data, focusing on the Eocene climatic optimum, the Oligocene glaciation, the Miocene optimum and the Pliocene.

## 3. Deep ocean temperature and sea level in the Cenozoic era

The *δ*^18^O stable isotope ratio was the first palaeothermometer, proposed by Urey [38] and developed especially by Emiliani [39]. There are now several alternative proxy measures of ancient climate change, but the *δ*^18^O data (figure 1*a*) of Zachos *et al.* [4], a conglomerate of the global ocean sediment cores, is well suited for our purpose as it covers the Cenozoic era with good temporal resolution. There are large, even dominant, non-climatic causes of *δ*^18^O changes over hundreds of millions of years [40], but non-climatic change may be small in the past few hundred million years [41] and is generally neglected in Cenozoic climate studies. The principal difficulty in using the *δ*^18^O record to estimate global deep ocean temperature, in the absence of non-climatic change, is that *δ*^18^O is affected by the global ice mass as well as the deep ocean temperature.

We make a simple estimate of global sea-level change for the Cenozoic era using the near-global *δ*^18^O compilation of Zachos *et al.* [4]. More elaborate and accurate approaches, including use of models, will surely be devised, but comparison of our result with other approaches is instructive regarding basic issues such as the vulnerability of today's ice sheets to near-term global warming and the magnitude of hysteresis effects in ice sheet growth and decay.

During the Early Cenozoic, between 65.5 and 35 Myr BP, the Earth was so warm that there was little ice on the planet and the deep ocean temperature is approximated by [6]
3.1
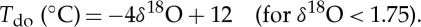
Hansen *et al.* [5] made the approximation that, as the Earth became colder and continental ice sheets grew, further increase in *δ*^18^O was due, in equal parts, to deep ocean temperature change and ice mass change,
3.2
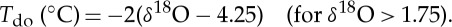
Equal division of the *δ*^18^O change into temperature change and ice volume change was suggested by comparing *δ*^18^O at the endpoints of the climate change from the nearly ice-free planet at 35 Myr BP (when *δ*^18^O approx. 1.75) with the Last Glacial Maximum (LGM), which peaked approximately 20 kyr BP. The change of *δ*^18^O between these two extreme climate states (approx. 3) is twice the change of *δ*^18^O due to temperature change alone (approx. 1.5), with the temperature change based on the linear relation (??eq3.1) and estimates of *T*_do_∼5^°^C at 35 Myr BP (figure 1) and approximately −1^°^C at the LGM [42].

This approximation can easily be made more realistic. Although ice volume and deep ocean temperature changes contributed comparable amounts to *δ*^18^O change on average over the full range from 35 Myr to 20 kyr BP, the temperature change portion of the *δ*^18^O change must decrease as the deep ocean temperature approaches the freezing point [43]. The rapid increase in *δ*^18^O in the past few million years was associated with the appearance of Northern Hemisphere ice sheets, symbolized by the dark blue bar in figure 1*a*.

The sea-level change between the LGM and Holocene was approximately 120 m [44,45]. Thus, two-thirds of the 180 m sea-level change between the ice-free planet and the LGM occurred with formation of Northern Hemisphere ice (and probably some increased volume of Antarctic ice). Thus, rather than taking the 180 m sea-level change between the nearly ice-free planet of 34 Myr BP and the LGM as being linear over the entire range (with 90 m for *δ*^18^O<3.25 and 90 m for *δ*^18^O>3.25), it is more realistic to assign 60 m of sea-level change to *δ*^18^O 1.75–3.25 and 120 m to *δ*^18^O>3.25. The total deep ocean temperature change of 6^°^C for the change of *δ*^18^O from 1.75 to 4.75 is then divided two-thirds (4^°^C) for the *δ*^18^O range 1.75–3.25 and 2^°^C for the *δ*^18^O range 3.25–4.75. Algebraically,
3.3
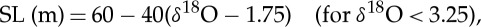

3.4
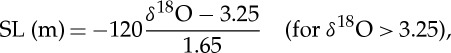

3.5
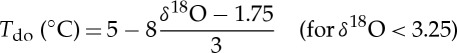

and
3.6
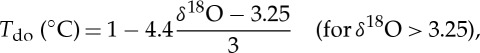
where SL is the sea level and its zero point is the Late Holocene level. The coefficients in equations (3.4) and (3.6) account for the fact that the mean LGM value of *δ*^18^O is approximately 4.9. The resulting deep ocean temperature is shown in figure 1*b* for the full Cenozoic era.

**Figure 2.**
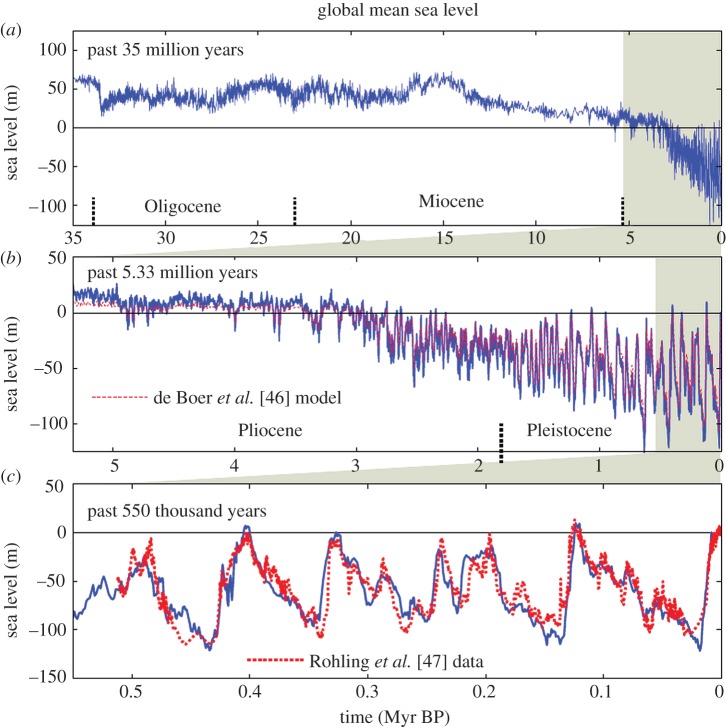
(*a*–*c*) Sea level from equations (3.3) and (3.4) using *δ*^18^O data of Zachos *et al.* [4], compared in (*b*) with ice sheet model results of de Boer *et al.* [46] and in (*c*) with the sea-level analysis of Rohling *et al.* [47].

**Figure 3.**
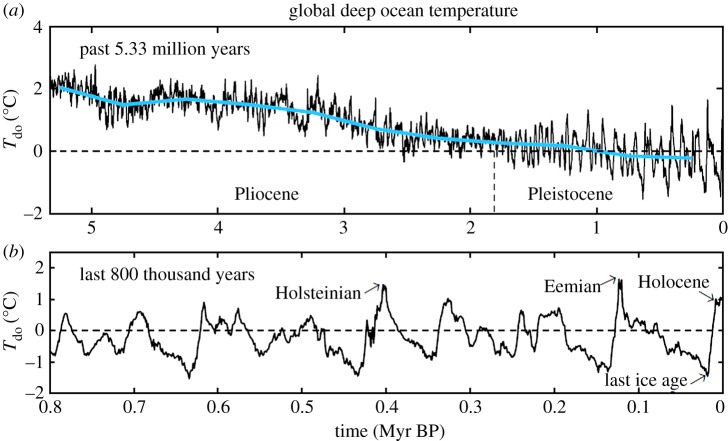
Deep ocean temperature in (*a*) the Pliocene and Pleistocene and (*b*) the last 800 000 years. High-frequency variations (black) are five-point running means of the original data [4], whereas the blue curve has a 500 kyr resolution. The deep ocean temperature for the entire Cenozoic era is in figure 1*b*.

Sea level from equations (3.3) and (3.4) is shown by the blue curves in figure 2, including comparison (figure 2*c*) with the Late Pleistocene sea-level record of Rohling *et al.* [47], which is based on analysis of Red Sea sediments, and comparison (figure 2*b*) with the sea-level chronology of de Boer *et al.* [46], which is based on ice sheet modelling with the *δ*^18^O data of Zachos *et al.* [4] as a principal input driving the ice sheet model. Comparison of our result with that of de Boer *et al.* [46] for the other periods of figure 2 is included in the electronic supplementary material, where we also make available our numerical data. Deep ocean temperature from equations (3.5) and (3.6) is shown for the Pliocene and Pleistocene in figure 3 and for the entire Cenozoic era in figure 1.

Differences between our inferred sea-level chronology and that from the ice sheet model [46] are relevant to the assessment of the potential danger to humanity from future sea-level rise. Our estimated sea levels have reached +5 to 10 m above the present sea level during recent interglacial periods that were barely warmer than the Holocene, whereas the ice sheet model yields maxima at most approximately 1 m above the current sea level. We find the Pliocene sea level varying between about +20 m and −50 m, with the Early Pliocene averaging about +15 m; the ice sheet model has a less variable sea level with the Early Pliocene averaging about +8 m. A 15 m sea-level rise implies that the East Antarctic ice sheet as well as West Antarctica and Greenland ice were unstable at a global temperature no higher than those projected to occur this century [1,48].

How can we interpret these differences, and what is the merit of our simple *δ*^18^O scaling? Ice sheet models constrained by multiple observations may eventually provide our best estimate of sea-level change, but as yet models are primitive. Hansen [49,50] argues that real ice sheets are more responsive to climate change than is found in most ice sheet models. Our simple scaling approximation implicitly assumes that ice sheets are sufficiently responsive to climate change that hysteresis is not a dominant effect; in other words, ice volume on millennial time scales is a function of temperature and does not depend much on whether the Earth is in a warming or cooling phase. Thus, our simple transparent calculation may provide a useful comparison with geological data for sea-level change and with results of ice sheet models.

We cannot *a priori* define accurately the error in our sea-level estimates, but we can compare with geological data in specific cases as a check on reasonableness. Our results (figure 2) yield two instances in the past million years when sea levels have reached heights well above the current sea level: +9.8 m in the Eemian (approx. 120 kyr BP, also known as Marine Isotope Stage 5e or MIS-5e) and +7.1 m in the Holsteinian (approx. 400 kyr BP, also known as MIS-11). Indeed, these are the two interglacial periods in the Late Pleistocene that traditional geological methods identify as probably having a sea level exceeding that in the Holocene. Geological evidence, mainly coral reefs on tectonically stable coasts, was described in the review of Overpeck *et al.* [51] as favouring an Eemian maximum of +4 to more than 6 m. Rohling *et al.* [52] cite many studies concluding that the mean sea level was 4–6 m above the current sea level during the warmest portion of the Eemian, 123–119 kyr BP; note that several of these studies suggest Eemian sea-level fluctuations up to +10 m, and provide the first continuous sea-level data supporting rapid Eemian sea-level fluctuations. Kopp *et al.* [53] made a statistical analysis of data from a large number of sites, concluding that there was a 95% probability that the Eemian sea level reached at least +6.6 m with a 67% probability that it exceeded 8 m.

The Holsteinian sea level is more difficult to reconstruct from geological data because of its age, and there has been a long-standing controversy concerning a substantial body of geological shoreline evidence for a +20 m Late Holsteinian sea level that Hearty and co-workers have found on numerous sites [54,55] (numerous pros and cons are contained in the references provided in our present paragraph). Rohling *et al.* [56] note that their temporally continuous Red Sea record ‘strongly supports the MIS-11 sea level review of Bowen [57], which also places MIS-11 sea level within uncertainties at the present-day level’. This issue is important because both ice core data [29] and ocean sediment core data (see below) indicate that the Holsteinian period was only moderately warmer than the Holocene with similar Earth orbital parameters. We suggest that the resolution of this issue is consistent with our estimate of the approximately +7 m Holsteinian global sea level, and is provided by Raymo & Mitrovica [58], who pointed out the need to make a glacial isostatic adjustment (GIA) correction for post-glacial crustal subsidence at the places where Hearty and others deduced local sea-level change. The uncertainties in GIA modelling led Raymo & Mitrovica [58] to conclude that the peak Holsteinian global sea level was in the range of +6 to 13 m relative to the present. Thus, it seems to us, there is a reasonable resolution of the long-standing Holsteinian controversy, with substantial implications for humanity, as discussed in later sections.

We now address differences between our sea-level estimates and those from ice sheet models. We refer to both the one-dimensional ice sheet modelling of de Boer *et al.* [46], which was used to calculate sea level for the entire Cenozoic era, and the three-dimensional ice sheet model of Bintanja *et al.* [59], which was used for simulations of the past million years. The differences most relevant to humanity occur in the interglacial periods slightly warmer than the Holocene, including the Eemian and Hosteinian, as well as the Pliocene, which may have been as warm as projected for later this century. Both the three-dimensional model of Bintanja *et al.* [59] and the one-dimensional model of de Boer *et al.* [46] yield maximum Eemian and Hosteinian sea levels of approximately 1 m relative to the Holocene. de Boer *et al.* [46] obtain approximately +8 m for the Early Pliocene, which compares with our approximately +15 m.

**Figure 4.**
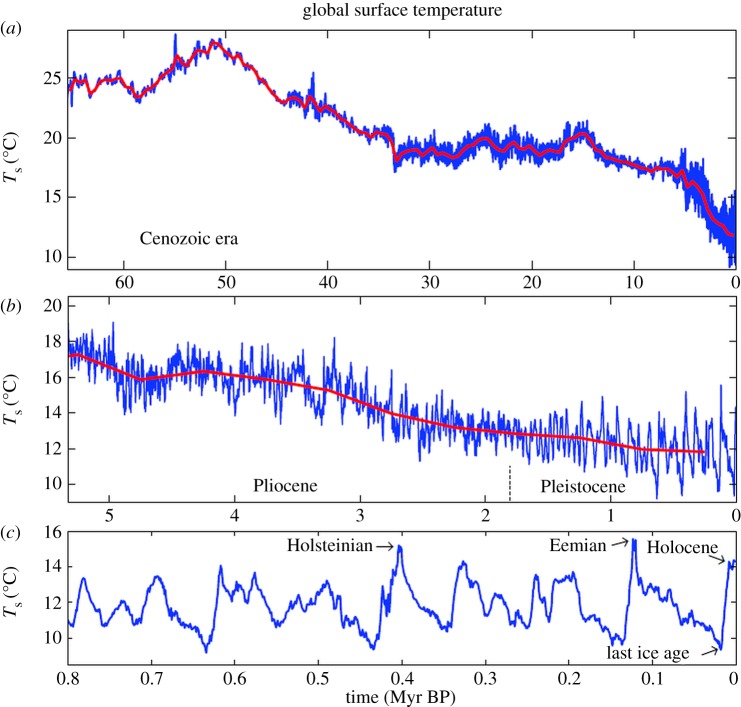
(*a*–*c*) Surface temperature estimate for the past 65.5 Myr, including an expanded time scale for (*b*) the Pliocene and Pleistocene and (*c*) the past 800 000 years. The red curve has a 500 kyr resolution. Data for this and other figures are available in the electronic supplementary material.

These differences reveal that the modelled ice sheets are less susceptible to change in response to global temperature variation than our *δ*^18^O analysis. Yet the ice sheet models do a good job of reproducing the sea-level change for climates colder than the Holocene, as shown in figure 2 and electronic supplementary material, figure S2. One possibility is that the ice sheet models are too lethargic for climates warmer than the Holocene. Hansen & Sato [60] point out the sudden change in the responsiveness of the ice sheet model of Bintanja *et al.* [59] when the sea level reaches today's level (figs 3 and 4 of Hansen & Sato [60]) and they note that the empirical sea-level data provide no evidence of such a sudden change. The explanation conceivably lies in the fact that the models have many parameters and their operation includes use of ‘targets’ [46] that affect the model results, because these choices might yield different results for warmer climates than the results for colder climates. Because of the potential that model development choices might be influenced by expectations of a ‘correct’ result, it is useful to have estimates independent of the models based on alternative assumptions.

Note that our approach also involves ‘targets’ based on expected behaviour, albeit simple transparent ones. Our two-legged linear approximation of the sea level (equations (3.3) and (3.4)) assumes that the sea level in the LGM was 120 m lower than today and that the sea level was 60 m higher than today 35 Myr BP. This latter assumption may need to be adjusted if glaciers and ice caps in the Eocene had a volume of tens of metres of sea level. However, Miller *et al.* [61] conclude that there was a sea level fall of approximately 55 m at the Eocene–Oligocene transition, consistent with our assumption that Eocene ice probably did not contain more than approximately 10 m of sea level.

Real-world data for the Earth's sea-level history ultimately must provide assessment of sea-level sensitivity to climate change. A recent comprehensive review [7] reveals that there are still wide uncertainties about the Earth's sea-level history that are especially large for time scales of tens of millions of years or longer, which is long enough for substantial changes in the shape and volume of ocean basins. Gasson *et al.* [7] plot regional (New Jersey) sea level (their fig. 14) against the deep ocean temperature inferred from the magnesium/calcium ratio (Mg/Ca) of deep ocean foraminifera [62], finding evidence for a nonlinear sea-level response to temperature roughly consistent with the modelling of de Boer *et al.* [46]. Sea-level change is limited for Mg/Ca temperatures up to about 5^°^C above current values, whereupon a rather abrupt sea-level rise of several tens of metres occurs, presumably representing the loss of Antarctic ice. However, the uncertainty in the reconstructed sea level is tens of metres and the uncertainty in the Mg/Ca temperature is sufficient to encompass the result from our *δ*^18^O prescription, which has comparable contributions of ice volume change and deep ocean temperature change at the Late Eocene glaciation of Antarctica.

Furthermore, the potential sea-level rise of most practical importance is the first 15 m above the Holocene level. It is such ‘moderate’ sea-level change for which we particularly question the projections implied by current ice sheet models. Empirical assessment depends upon real-world sea-level data in periods warmer than the Holocene. There is strong evidence, discussed above, that the sea level was several metres higher in recent warm interglacial periods, consistent with our data interpretation. The Pliocene provides data extension to still warmer climates. Our interpretation of *δ*^18^O data suggests that Early Pliocene sea-level change (due to ice volume change) reached about +15 m, and it also indicates sea-level fluctuations as large as 20–40 m. Sea-level data for Mid-Pliocene warm periods, of comparable warmth to average Early Pliocene conditions (figure 3), suggest sea heights as great as +15–25 m [63,64]. Miller *et al.* [61] find a Pliocene sea-level maximum of 22±10 m (95% confidence). GIA creates uncertainty in sea-level reconstructions based on shoreline geological data [65], which could be reduced via appropriately distributed field studies. Dwyer & Chandler [64] separate Pliocene ice volume and temperature in deep ocean *δ*^18^O via ostracode Mg/Ca temperatures, finding sea-level maxima and oscillations comparable to our results. Altogether, the empirical data provide strong evidence against the lethargy and strong hysteresis effects of at least some ice sheet models.

## 4. Surface air temperature change

The temperature of most interest to humanity is the surface air temperature. A record of past global surface temperature is required for empirical inference of global climate sensitivity. Given that climate sensitivity can depend on the initial climate state and on the magnitude and sign of the climate forcing, a continuous record of global temperature over a wide range of climate states would be especially useful. Because of the singularly rich climate story in Cenozoic deep ocean *δ*^18^O (figure 1), unrivalled in detail and self-consistency by alternative climate proxies, we use deep ocean *δ*^18^O to provide the fine structure of Cenozoic temperature change. We use surface temperature proxies from the LGM, the Pliocene and the Eocene to calibrate and check the relation between deep ocean and surface temperature change.

The temperature signal in deep ocean *δ*^18^O refers to the sea surface where cold dense water formed and sank to the ocean bottom, the principal location of deep water formation being the Southern Ocean. Empirical data and climate models concur that surface temperature change is generally amplified at high latitudes, which tends to make temperature change at the site of deep water formation an overestimate of global temperature change. Empirical data and climate models also concur that surface temperature change is amplified over land areas, which tends to make temperature change at the site of deep water an underestimate of the global temperature. Hansen *et al.* [5] and Hansen & Sato [60] noted that these two factors were substantially offsetting, and thus they made the assumption that benthic foraminifera provide a good approximation of global mean temperature change for most of the Cenozoic era.

However, this approximation breaks down in the Late Cenozoic for two reasons. First, the deep ocean and high-latitude surface ocean where deep water forms are approaching the freezing point in the Late Cenozoic. As the Earth's surface cools further, cold conditions spread to lower latitudes but polar surface water and the deep ocean cannot become much colder, and thus the benthic foraminifera record a temperature change smaller than the global average surface temperature change [43]. Second, the last 5.33 Myr of the Cenozoic, the Pliocene and Pleistocene, was the time that global cooling reached a degree such that large ice sheets could form in the Northern Hemisphere. When a climate forcing, or a slow climate feedback such as ice sheet formation, occurs in one hemisphere, the temperature change is much larger in the hemisphere with the forcing (cf. examples in Hansen *et al.* [66]). Thus, cooling during the last 5.33 Myr in the Southern Ocean site of deep water formation was smaller than the global average cooling.

We especially want our global surface temperature reconstruction to be accurate for the Pliocene and Pleistocene because the global temperature changes that are expected by the end of this century, if humanity continues to rapidly change atmospheric composition, are of a magnitude comparable to climate change in those epochs [1,48]. Fortunately, sufficient information is available on surface temperature change in the Pliocene and Pleistocene to allow us to scale the deep ocean temperature change by appropriate factors, thus retaining the temporal variations in the *δ*^18^O while also having a realistic magnitude for the total temperature change over these epochs.

Pliocene temperature is known quite well because of a long-term effort to reconstruct the climate conditions during the Mid-Pliocene warm period (3.29–2.97 Myr BP) and a coordinated effort to numerically simulate the climate by many modelling groups ([67] and papers referenced therein). The reconstructed Pliocene climate used data for the warmest conditions found in the Mid-Pliocene period, which would be similar to average conditions in the Early Pliocene (figure 3). These boundary conditions were used by eight modelling groups to simulate Pliocene climate with atmospheric general circulation models. Although atmosphere–ocean models have difficulty replicating Pliocene climate, atmospheric models forced by specified surface boundary conditions are expected to be capable of calculating global surface temperature with reasonable accuracy. The eight global models yield Pliocene global warming of 3±1^°^C relative to the Holocene [68]. This Pliocene warming is an amplification by a factor of 2.5 of the deep ocean temperature change.

Similarly, for the reasons given above, the deep ocean temperature change of 2.25^°^C between the Holocene and the LGM is surely an underestimate of the surface air temperature change. Unfortunately, there is a wide range of estimates for LGM cooling, approximately 3–6^°^C, as discussed in §6. Thus, we take 4.5^°^C as our best estimate for LGM cooling, implying an amplification of surface temperature change by a factor of two relative to deep ocean temperature change for this climate interval.

We obtain an absolute temperature scale using the Jones *et al.* [69] estimate of 14^°^C as the global mean surface temperature for 1961–1990, which corresponds to approximately 13.9^°^C for the 1951–1980 base period that we normally use [70] and approximately 14.4^°^C for the first decade of the twenty-first century. We attach the instrumental temperature record to the palaeo data by assuming that the first decade of the twenty-first century exceeds the Holocene mean by 0.25±0.25^°^C. Global temperature probably declined over the past several millennia [71], but we suggest that warming of the past century has brought global temperature to a level that now slightly exceeds the Holocene mean, judging from sea-level trends and ice sheet mass loss. Sea level is now rising 3.1 mm per year or 3.1 m per millennium [72], an order of magnitude faster than the rate during the past several thousand years, and Greenland and Antarctica are losing mass at accelerating rates [73,74]. Our assumption that global temperature passed the Holocene mean a few decades ago is consistent with the rapid change of ice sheet mass balance in the past few decades [75]. The above concatenation of instrumental and palaeo records yields a Holocene mean of 14.15^°^C and Holocene maximum (from five-point smoothed *δ*^18^O) of 14.3^°^C at 8.6 kyr BP.

Given a Holocene temperature of 14.15^°^C and LGM cooling of 4.5^°^C, the Early Pliocene mean temperature 3^°^C warmer than the Holocene leads to the following prescription:
4.1
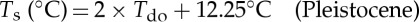
and
4.2
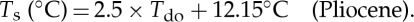
This prescription yields a maximum Eemian temperature of 15.56^°^C, which is approximately 1.4^°^C warmer than the Holocene mean and approximately 1.8^°^C warmer than the 1880–1920 mean. Clark & Huybers [76] fit a polynomial to proxy temperatures for the Eemian, finding warming as much as +5^°^C at high northern latitudes but global warming of +1.7^°^C ‘relative to the present interglacial before industrialization’. Other analyses of Eemian data find global sea surface temperature warmer than the Late Holocene by 0.7±0.6^°^C [77] and all-surface warming of 2^°^C [78], all in reasonable accord with our prescription.

Our first estimate of global temperature for the remainder of the Cenozoic assumes that *ΔT*_s_=*ΔT*_do_ prior to 5.33 Myr BP, i.e. prior to the Plio-Pleistocene, which yields a peak *T*_s_ of approximately 28^°^C at 50 Myr BP (figure 4). This is at the low end of the range of current multi-proxy measures of sea surface temperature for the Early Eocene Climatic Optimum (EECO) [79–81]. Climate models are marginally able to reproduce this level of Eocene warmth, but the models require extraordinarily high CO_2_ levels, for example 2240–4480 ppm [82] and 2500–6500 ppm [83], and the quasi-agreement between data and models requires an assumption that some of the proxy temperatures are biased towards summer values. Moreover, taking the proxy sea surface temperature data for the peak Eocene period (55–48 Myr BP) at face value yields a global temperature of 33–34^°^C (fig. 3 of Bijl *et al.* [84]), which would require an even larger CO_2_ amount with the same climate models. Thus, below we also consider the implications for climate sensitivity of an assumption that *ΔT*_s_=1.5×*ΔT*_do_ prior to 5.33 Myr BP, which yields *T*_s_ approximately 33^°^C at 50 Myr BP (see electronic supplementary material, figure S3).

## 5. Climate sensitivity

Climate sensitivity (*S*) is the equilibrium global surface temperature change (*ΔT*_eq_) in response to a specified unit forcing after the planet has come back to energy balance,
5.1
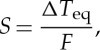
i.e. climate sensitivity is the eventual (equilibrium) global temperature change per unit forcing. Climate sensitivity depends upon climate feedbacks, the many physical processes that come into play as climate changes in response to a forcing. Positive (amplifying) feedbacks increase the climate response, whereas negative (diminishing) feedbacks reduce the response.

We usually discuss climate sensitivity in terms of a global mean temperature response to a 4 W m^−2^ CO_2_ forcing. One merit of this standard forcing is that its magnitude is similar to an anticipated near-term human-made climate forcing, thus avoiding the need to continually scale the unit sensitivity to achieve an applicable magnitude. A second merit is that the efficacy of forcings varies from one forcing mechanism to another [66]; so it is useful to use the forcing mechanism of greatest interest. Finally, the 4 W m^−2^ CO_2_ forcing avoids the uncertainty in the exact magnitude of a doubled CO_2_ forcing [1,48] estimate of 3.7 W m^−2^ for doubled CO_2_, whereas Hansen *et al.* [66] obtain 4.1 W m^−2^, as well as problems associated with the fact that a doubled CO_2_ forcing varies as the CO_2_ amount changes (the assumption that each CO_2_ doubling has the same forcing is meant to approximate the effect of CO_2_ absorption line saturation, but actually the forcing per doubling increases as CO_2_ increases [66,85]).

Climate feedbacks are the core of the climate problem. Climate feedbacks can be confusing, because in climate analyses what is sometimes a climate forcing is at other times a climate feedback. A CO_2_ decrease from, say, approximately 1000 ppm in the Early Cenozoic to 170–300 ppm in the Pleistocene, caused by shifting plate tectonics, is a climate forcing, a perturbation of the Earth's energy balance that alters the temperature. Glacial–interglacial oscillations of the CO_2_ amount and ice sheet size are both slow climate feedbacks, because glacial–interglacial climate oscillations largely are instigated by insolation changes as the Earth's orbit and tilt of its spin axis change, with the climate change then amplified by a nearly coincident change of the CO_2_ amount and the surface albedo. However, for the sake of analysis, we can also choose and compare periods that are in quasi-equilibrium, periods during which there was little change of the ice sheet size or the GHG amount. For example, we can compare conditions averaged over several millennia in the LGM with mean Holocene conditions. The Earth's average energy imbalance within each of these periods had to be a small fraction of 1 W m^−2^. Such a planetary energy imbalance is very small compared with the boundary condition ‘forcings’, such as changed GHG amount and changed surface albedo that maintain the glacial-to-interglacial climate change.

### (a) Fast-feedback sensitivity: Last Glacial Maximum–Holocene

The average fast-feedback climate sensitivity over the LGM–Holocene range of climate states can be assessed by comparing estimated global temperature change and climate forcing change between those two climate states [3,86]. The appropriate climate forcings are the changes in long-lived GHGs and surface properties on the planet. Fast feedbacks include water vapour, clouds, aerosols and sea ice changes.

This fast-feedback sensitivity is relevant to estimating the climate impact of human-made climate forcings, because the size of ice sheets is not expected to change significantly in decades or even in a century and GHGs can be specified as a forcing. GHGs change in response to climate change, but it is common to include these feedbacks as part of the climate forcing by using observed GHG changes for the past and calculated GHGs for the future, with calculated amounts based on carbon cycle and atmospheric chemistry models.

**Figure 5.**
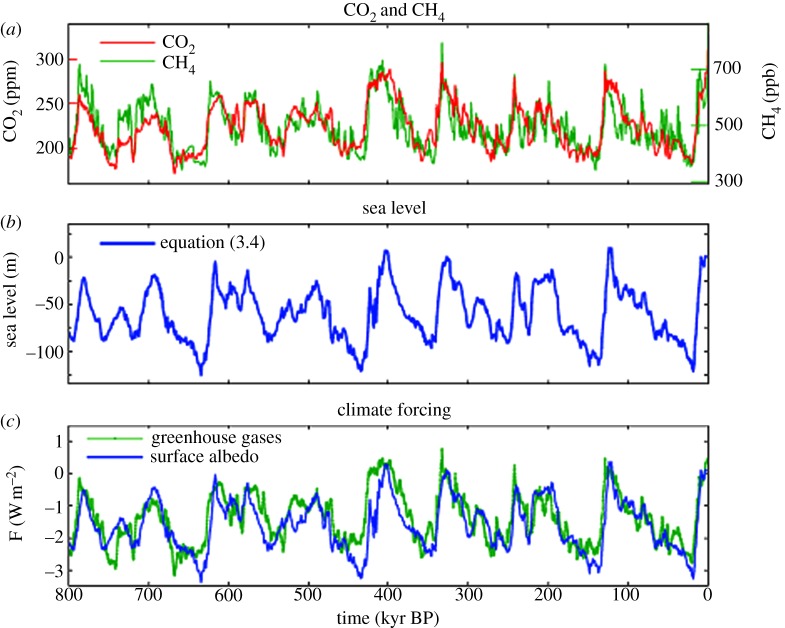
(*a*) CO_2_ and CH_4_ from ice cores; (*b*) sea level from equation (3.4) and (*c*) resulting climate forcings (see text).

Climate forcings due to past changes in GHGs and surface albedo can be computed for the past 800 000 years using data from polar ice cores and ocean sediment cores. We use CO_2_ [87] and CH_4_ [88] data from Antarctic ice cores (figure 5*a*) to calculate an effective GHG forcing as follows:
5.2
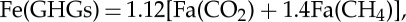
where Fa is the adjusted forcing, i.e. the planetary energy imbalance due to the GHG change after the stratospheric temperature has time to adjust to the gas change. Fe, the effective forcing, accounts for variable efficacies of different climate forcings [66]. Formulae for Fa of each gas are given by Hansen *et al.* [89]. The factor 1.4 converts the adjusted forcing of CH_4_ to its effective forcing, Fe, which is greater than Fa mainly because of the effect of CH_4_ on the tropospheric ozone and the stratospheric water vapour [66]. The factor 1.12 approximates the forcing by N_2_O changes, which are not as well preserved in the ice cores but have a strong positive correlation with CO_2_ and CH_4_ changes [90]. The factor 1.12 is smaller than the 1.15 used by Hansen *et al.* [91], and is consistent with estimates of the N_2_O forcing in the current Goddard Institute for Space Studies (GISS) radiation code and that of the Intergovernmental Panel on Climate Change (IPCC) [1,48]. Our LGM–Holocene GHG forcing (figure 5*c*) is approximately 3 m^−2^, moderately larger than the 2.8 W m^−2^ estimated by IPCC [1,48] because of our larger effective CH_4_ forcing.

Climate forcing due to surface albedo change is a function mainly of the sea level, which implicitly defines ice sheet size. Albedo change due to LGM–Holocene vegetation change, much of which is inherent with ice sheet area change, and albedo change due to coastline movement are lumped together with ice sheet area change in calculating the surface albedo climate forcing. An ice sheet forcing does not depend sensitively on the ice sheet shape or on how many ice sheets the ice volume is divided among and is nearly linear in sea-level change (see electronic supplementary material, figure S4, and [5]). For the sake of simplicity, we use the linear relation in Hansen *et al.* [5] and electronic supplementary material, figure S4; thus, 5 W m^−2^ between the LGM and ice-free conditions and 3.4 W m^−2^ between the LGM and Holocene. This scale factor was based on simulations with an early climate model [3,92]; comparable forcings are found in other models (e.g. see discussion in [93]), but results depend on cloud representations, assumed ice albedo and other factors; so the uncertainty is difficult to quantify. We subjectively estimate an uncertainty of approximately 20%.

**Figure 6.**
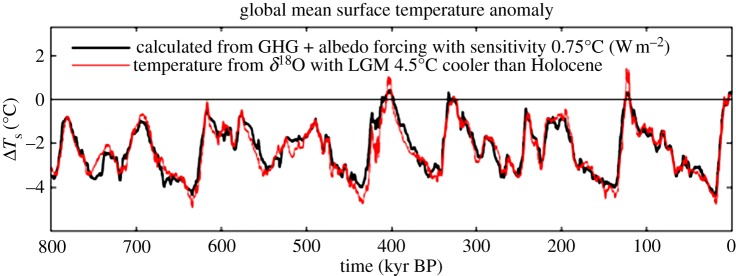
Calculated surface temperature for forcings of figure 5*c* with a climate sensitivity of 0.75^°^C per W m^−2^, compared with 2×*ΔT*_do_. Zero point is the Holocene (10 kyr) mean.

Global temperature change obtained by multiplying the sum of the two climate forcings in figure 5*c* by a sensitivity of 3/4^°^C per W m^−2^ yields a remarkably good fit to ‘observations’ (figure 6), where the observed temperature is 2×*ΔT*_do_, with 2 being the scale factor required to yield the estimated 4.5^°^C LGM–Holocene surface temperature change. The close match is partly a result of the fact that sea-level and temperature data are derived from the same deep ocean record, but use of other sea-level reconstructions still yields a good fit between the calculated and observed temperature [5]. However, exactly the same match as in figure 6 is achieved with a fast-feedback sensitivity of 1^°^C per W m^−2^ if the LGM cooling is 6^°^C or with a sensitivity of 0.5^°^C per W m^−2^ if the LGM cooling is 3^°^C.

Accurate data defining LGM–Holocene warming would aid empirical evaluation of fast-feedback climate sensitivity. Remarkably, the range of recent estimates of LGM–Holocene warming, from approximately 3^°^C [94] to approximately 6^°^C [95], is about the same as at the time of the CLIMAP [96] project. Given today's much improved analytic capabilities, a new project to define LGM climate conditions, analogous to the Pliocene Research, Interpretation and Synoptic Mapping (PRISM) Pliocene data reconstruction [97,98] and Pliocene Model Intercomparison Project (PlioMIP) model intercomparisons [67,68], could be beneficial. In §7*b*, we suggest that a study of Eemian glacial–interglacial climate change could be even more definitive. Combined LGM, Eemian and Pliocene studies would address an issue raised at a recent workshop [99]: the need to evaluate how climate sensitivity varies as a function of the initial climate state. The calculations below were initiated after the workshop as another way to address that question.

### (b) Fast-feedback sensitivity: state dependence

Climate sensitivity must be a strong function of the climate state. Simple climate models show that, when the Earth becomes cold enough for the ice cover to approach the tropics, the amplifying albedo feedback causes rapid ice growth to the Equator: ‘snowball Earth’ conditions [100]. Real-world complexity, including ocean dynamics, can mute this sharp bifurcation to a temporarily stable state [101], but snowball events have occurred several times in the Earth's history when the younger Sun was dimmer than today [102]. The Earth escaped snowball conditions owing to limited weathering in that state, which allowed volcanic CO_2_ to accumulate in the atmosphere until there was enough CO_2_ for the high sensitivity to cause rapid deglaciation [103].

Climate sensitivity at the other extreme, as the Earth becomes hotter, is also driven mainly by an H_2_O feedback. As climate forcing and temperature increase, the amount of water vapour in the air increases and clouds may change. Increased water vapour makes the atmosphere more opaque in the infrared region that radiates the Earth's heat to space, causing the radiation to emerge from higher colder layers, thus reducing the energy emitted to space. This amplifying feedback has been known for centuries and was described remarkably well by Tyndall [104]. Ingersoll [105] discussed the role of water vapours in the ‘runaway greenhouse effect’ that caused the surface of Venus to eventually become so hot that carbon was ‘baked’ from the planet's crust, creating a hothouse climate with almost 100 bars of CO_2_ in the air and a surface temperature of about 450^°^C, a stable state from which there is no escape. Arrival at this terminal state required passing through a ‘moist greenhouse’ state in which surface water evaporates, water vapour becomes a major constituent of the atmosphere and H_2_O is dissociated in the upper atmosphere with the hydrogen slowly escaping to space [106]. That Venus had a primordial ocean, with most of the water subsequently lost to space, is confirmed by the present enrichment of deuterium over ordinary hydrogen by a factor of 100 [107], the heavier deuterium being less efficient in escaping gravity to space.

The physics that must be included to investigate the moist greenhouse is principally: (i) accurate radiation incorporating the spectral variation of gaseous absorption in both the solar radiation and thermal emission spectral regions, (ii) atmospheric dynamics and convection with no specifications favouring artificial atmospheric boundaries, such as between a troposphere and stratosphere, (iii) realistic water vapour physics, including its effect on atmospheric mass and surface pressure, and (iv) cloud properties that respond realistically to climate change. Conventional global climate models are inappropriate, as they contain too much other detail in the form of parametrizations or approximations that break down as climate conditions become extreme.

We use the simplified atmosphere–ocean model of Russell *et al.* [108], which solves the same fundamental equations (conservation of energy, momentum, mass and water substance, and the ideal gas law) as in more elaborate global models. Principal changes in the physics in the current version of the model are use of a step-mountain C-grid atmospheric vertical coordinate [109], addition of a drag in the grid-scale momentum equation in both atmosphere and ocean based on subgrid topography variations, and inclusion of realistic ocean tides based on exact positioning of the Moon and Sun. Radiation is the *k*-distribution method of Lacis & Oinas [110] with 25 *k*-values; the sensitivity of this specific radiation code is documented in detail by Hansen *et al.* [111]. Atmosphere and ocean dynamics are calculated on 3^°^×4^°^ Arakawa C-grids. There are 24 atmospheric layers. In our present simulations, the ocean's depth is reduced to 100 m with five layers so as to achieve a rapid equilibrium response to forcings; this depth limitation reduces poleward ocean transport by more than half. Moist convection is based on a test of moist static stability as in Hansen *et al.* [92]. Two cloud types occur: moist convective clouds, when the atmosphere is moist statically unstable, and large-scale super-saturation, with cloud optical properties based on the amount of moisture removed to eliminate super-saturation, with scaling coefficients chosen to optimize the control run's fit with global observations [108,112]. To avoid long response times in extreme climates, today's ice sheets are assigned surface properties of the tundra, thus allowing them to have a high albedo snow cover in cold climates but darker vegetation in warm climates. The model, the present experiments and more extensive experiments will be described in a forthcoming paper [112].

**Figure 7.**
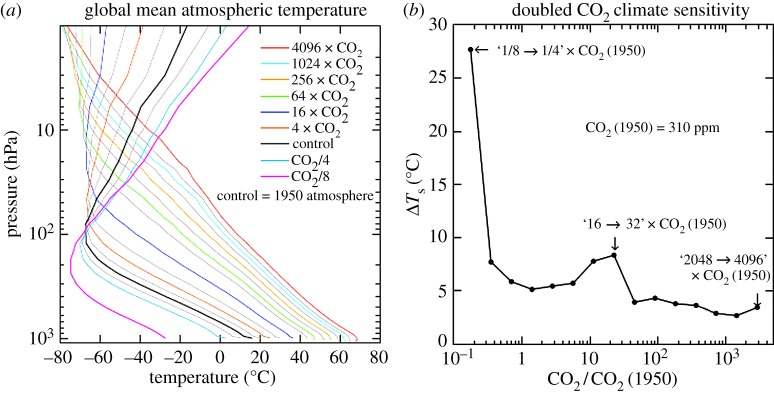
(*a*) The calculated global mean temperature for successive doublings of CO_2_ (legend identifies every other case) and (*b*) the resulting climate sensitivity (1×CO_2_=310 ppm).

The equilibrium response of the control run (1950 atmospheric composition, CO_2_ approx. 310 ppm) and runs with successive CO_2_ doublings and halvings reveals that snowball Earth instability occurs just beyond three CO_2_ halvings. Given that a CO_2_ doubling or halving is equivalent to a 2% change in solar irradiance [66] and the estimate that solar irradiance was approximately 6% lower 600 Ma at the most recent snowball Earth occurrence [113], figure 7 implies that about 300 ppm CO_2_ or less was sufficiently small to initiate glaciation at that time.

Climate sensitivity reaches large values at 8–32×CO_2_ (approx. 2500–10 000 ppm; figure 7*b*). High sensitivity is caused by increasing water vapour as the tropopause rises and diminishing low cloud cover, but the sensitivity decreases for still larger CO_2_ as cloud optical thickness and planetary albedo increase, as shown by Russell *et al.* [112]. The high sensitivity for CO_2_ less than 4×CO_2_ is due partly to the nature of the experiments (Greenland and Antarctic ice sheets being replaced by the tundra). High albedo snow cover on these continents largely disappears between 1×CO_2_ and 4×CO_2_, thus elevating the calculated fast-feedback sensitivity from approximately 4^°^C to approximately 5^°^C. In the real world, we would expect the Greenland and Antarctic ice sheets to be nearly eliminated and replaced by partially vegetated surfaces already at 2×CO_2_ (620 ppm) equilibrium climate. In other words, if the Greenland/Antarctic surface albedo change were identified as a slow feedback, rather than as a fast-feedback snow effect as it is in figure 7, the fast-feedback sensitivity at 1–4×CO_2_ would be approximately 4^°^C. Thus, the sensitivity approximately 8^°^C per CO_2_ doubling in the range of 8–32×CO_2_ is a very large increase over sensitivity at smaller CO_2_ amounts.

How confident are we in the modelled fast-feedback sensitivity (figure 7*b*)? We suspect that the modelled water vapour feedback may be moderately exaggerated, because the water vapour amount in the control run exceeds observed amounts. In addition, the area of sea ice in the control run exceeds observations, which may increase the modelled sensitivity in the 1–4×CO_2_ range. On the other hand, we probably underestimate the sensitivity at very high CO_2_ amounts, because our model (such as most climate models) does not change the total atmospheric mass as the CO_2_ amount varies. Mass change due to conceivable fossil fuel loading (up to say 16×CO_2_) is unlikely to have much effect, but sensitivity is probably underestimated at high CO_2_ amounts owing to self-broadening of CO_2_ absorption lines. The increased atmospheric mass is also likely to alter the cloud feedback, which otherwise is a strongly diminishing feedback at very large CO_2_ amounts. Atmospheric mass will be important after the Earth has lost its ocean and carbon is baked into the atmosphere. These issues are being examined by Russell *et al.* [112].

Earth today, with approximately 1.26 times 1950 CO_2_, is far removed from the snowball state. Because of the increase in solar irradiance over the past 600 Myr and volcanic emissions, no feasible CO_2_ amount could take the Earth back to snowball conditions. Similarly, a Venus-like baked-crust CO_2_ hothouse is far distant because it cannot occur until the ocean escapes to space. We calculate an escape time of the order of 10^8^–10^9^ years even with the increased stratospheric water vapour and temperature at 16×CO_2_. Given the transient nature of a fossil fuel CO_2_ injection, the continuing forcing required to achieve a terminal Venus-like baked-crust CO_2_ hothouse must wait until the Sun's brightness has increased on the billion year time scale. However, the planet could become uninhabitable long before that.

The practical concern for humanity is the high climate sensitivity and the eventual climate response that may be reached if all fossil fuels are burned. Estimates of the carbon content of all fossil fuel reservoirs including unconventional fossil fuels such as tar sands, tar shale and various gas reservoirs that can be tapped with developing technology [114] imply that CO_2_ conceivably could reach a level as high as 16 times the 1950 atmospheric amount. In that event, figure 7 suggests a global mean warming approaching 25^°^C, with much larger warming at high latitudes (see electronic supplementary material, figure S6). The result would be a planet on which humans could work and survive outdoors in the summer only in mountainous regions [115,116]—and there they would need to contend with the fact that a moist stratosphere would have destroyed the ozone layer [117].

## 6. Earth system sensitivity

GHG and surface albedo changes, which we treated as specified climate forcings in evaluating fast-feedback climate sensitivity, are actually slow climate feedbacks during orbit-instigated Pleistocene glacial–interglacial climate swings. Given that GHG and albedo feedbacks are both strong amplifying feedbacks, indeed accounting by themselves for most of the global Pleistocene climate variation, it is apparent that today's climate sensitivity on millennial time scales must be substantially larger than the fast-feedback sensitivity.

Climate sensitivity including slow feedbacks is described as ‘Earth system sensitivity’ [118–120]. There are alternative choices for the feedbacks included in Earth system sensitivity. Hansen & Sato [60] suggest adding slow feedbacks one by one, creating a series of increasingly comprehensive Earth system climate sensitivities; specifically, they successively move climate-driven changes in surface albedo, non-CO_2_ GHGs and CO_2_ into the feedback category, at which point the Earth system sensitivity is relevant to an external forcing such as changing solar irradiance or human-made forcings. At each level, in this series, the sensitivity is state dependent.

Our principal aim here is to use Cenozoic climate change to infer information on the all-important fast-feedback climate sensitivity, including its state dependence, via analysis of Earth system sensitivity. CO_2_ is clearly the dominant forcing of the long-term Cenozoic cooling, in view of the abundant evidence that CO_2_ reached levels of the order of 1000 ppm in the Early Cenozoic [9], as discussed in the Overview above. Thus, our approach is to examine Earth system sensitivity to CO_2_ change by calculating the CO_2_ history required to produce our reconstructed Cenozoic temperature history for alternative state-independent and state-dependent climate sensitivities. By comparing the resulting CO_2_ histories with CO_2_ proxy data, we thus assess the most realistic range for climate sensitivity.

Two principal uncertainties in this analysis are (i) global temperature at the EECO approximately 50 Myr BP and (ii) CO_2_ amount at that time. We use EECO approximately 28^°^C (figure 4) as our standard case, but we repeat the analysis with EECO approximately 33^°^C (see electronic supplementary material, figure S3), thus allowing inference of how the conclusions change if knowledge of Eocene temperature changes.

Similarly, our graphs allow the inferred climate sensitivity to be adjusted if improved knowledge of CO_2_ 50 Myr BP indicates a value significantly different from approximately 1000 ppm.

To clarify our calculations, let us first assume that fast-feedback climate sensitivity is a constant (state-independent) 3^°^C for doubled CO_2_ (0.75^°^C per W m^−2^). It is then trivial to convert our global temperature for the Cenozoic (figure 4*a*) to the total climate forcing throughout the Cenozoic, which is shown in the electronic supplementary material, figure S4*a*, as are results of subsequent steps. Next, we subtract the solar forcing, a linear increase of 1 W m^−2^ over the Cenozoic era due to the Sun's 0.4% irradiance increase [121], and the surface albedo forcing due to changing ice sheet size, which we take as linear at 5 W m^−2^ for the 180 m sea-level change from 35 Myr BP to the LGM. These top-of-the-atmosphere and surface forcings are moderate in size, compared with the total forcing over the Cenozoic, and partially offsetting, as shown in the electronic supplementary material, figure S4*b*. The residual forcing, which has a maximum of approximately 17 W m^−2^ just prior to 50 Myr BP, is the atmospheric forcing due to GHGs. Non-CO_2_ GHGs contribute 25% of the total GHG forcing in the period of ice core measurements. Atmospheric chemistry simulations [122] reveal continued growth of non-CO_2_ gases (N_2_O, CH_4_ and tropospheric O_3_) in warmer climates, at only a slightly lower rate (1.7–2.3 W m^−2^ for 4×CO_2_, which itself is approx. 8 W m^−2^). Thus, we take the CO_2_ forcing as 75% of the GHG forcing throughout the Cenozoic in our standard case, but we also consider the extreme case in which non-CO_2_ gases are fixed and thus contribute no climate forcing.

**Figure 8.**
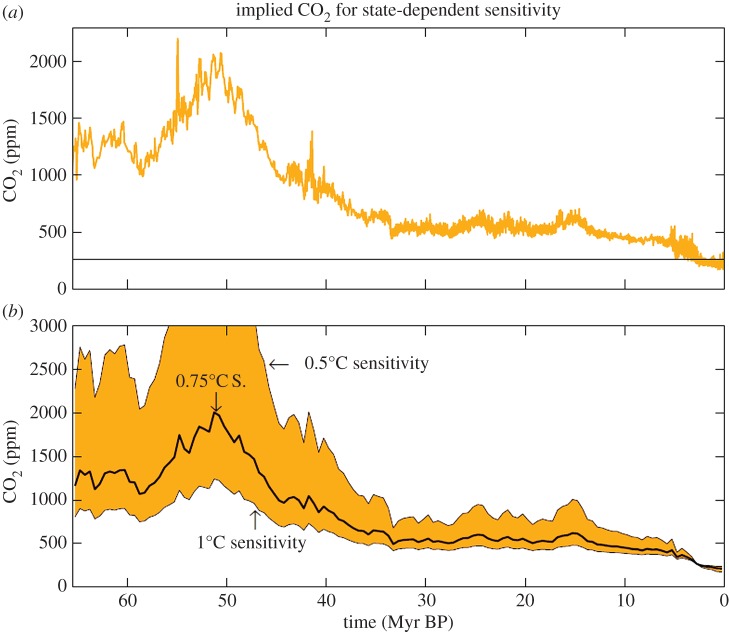
(*a*) CO_2_ amount required to yield a global temperature of figure 4*a* if fast-feedback climate sensitivity is 0.75^°^C per W m^−2^ and non-CO_2_ GHGs contribute 25% of the GHG forcing. (*b*) Same as in (*a*), but with temporal resolution 0.5 Myr and for three choices of fast-feedback sensitivity; the CO_2_ peak exceeds 5000 ppm in the case of 0.5^°^C sensitivity. The horizontal line is the Early–Mid-Holocene 260 ppm CO_2_ level.

A CO_2_ forcing is readily converted to the CO_2_ amount; we use the equation in table 1 of Hansen *et al.* [89]. The resulting Cenozoic CO_2_ history required to yield the global surface temperature of figure 4*a* is shown in figure 8*a* for state-independent climate sensitivity with non-CO_2_ GHGs providing 25% of the GHG climate forcing. The peak CO_2_ in this case is approximately 2000 ppm. If non-CO_2_ GHGs provide less than 25% of the total GHG forcing, then the inferred CO_2_ amount would be even greater. Results for alternative sensitivities, as in figure 8*b*, are calculated for a temporal resolution of 0.5 Myr to smooth out glacial–interglacial CO_2_ oscillations, as our interest here is in CO_2_ as a climate forcing.

We focus on the CO_2_ amount 50 Myr BP averaged over a few million years in assessing the realism of our inferred CO_2_ histories, because CO_2_ variations in the Cenozoic remain very uncertain despite the success of Beerling & Royer [9] in eliminating the most extreme outliers. Beerling & Royer [9] find a best-fit CO_2_ at 50 Myr BP of about 1000 ppm—see their figure 1, which also indicates that CO_2_ at 50 Myr BP was almost certainly in the range of 750–1500 ppm, even though it is impossible to provide a rigorous confidence interval.

We conclude that the average fast-feedback climate sensitivity during the Cenozoic is larger than the canonical 3^°^C for 2×CO_2_ (0.75^°^C per W m^−2^) that has long been the central estimate for current climate. An average 4^°^C for 2×CO_2_ (1^°^C per W m^−2^) provides a good fit to the target 1000 ppm CO_2_, but the sensitivity must be still higher if non-CO_2_ GHG forcings amplify the CO_2_ by less than one-third, i.e. provide less than 25% of the total GHG forcing.

### (a) State-dependent climate sensitivity

More realistic assessment should account for the state dependence of climate sensitivity. Thus, we make the same calculations for the state-dependent climate sensitivity of the Russell climate model, i.e. we use the fast-feedback climate sensitivity of figure 7*b*. In addition, for the purpose of assessing how the results depend upon climate sensitivity, we consider a second case in which we reduce the Russell sensitivity of figure 7*b* by the factor two-thirds.

**Figure 9.**
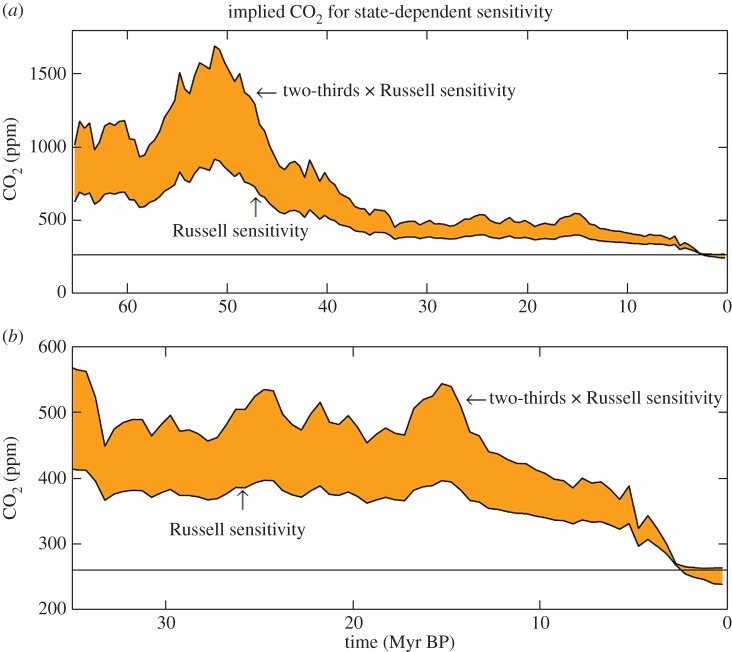
(*a*) CO_2_ amount required to yield the global temperature history of figure 4*a* if fast-feedback climate sensitivity is that calculated with the Russell model, i.e. the sensitivity shown in figure 7*b*, and two-thirds of that sensitivity. These results assume that non-CO_2_ GHGs provide 25% of the GHG climate forcing. (*b*) Vertical expansion for the past 35 Myr.

The estimated 1000 ppm of CO_2_ at 50 Myr BP falls between the Russell sensitivity and two-thirds of the Russell sensitivity, though closer to the full Russell sensitivity. If the non-CO_2_ GHG forcing is less than one-third of the CO_2_ forcing, the result is even closer to the full Russell sensitivity. With these comparisons at 50 Myr BP in mind, we can use figure 9 to infer the likely CO_2_ amount at other times. The End-Eocene transition began at about 500 ppm and fell to about 400 ppm. The Mid-Miocene warmth, which peaked at about 15 Myr BP, required a CO_2_ increase of only a few tens of ppm with the Russell sensitivity, but closer to 100 ppm if the true sensitivity is only two-thirds of the Russell sensitivity. The higher (full Russell) sensitivity requires much less CO_2_ change to produce the Mid-Miocene warming for two reasons: (i) the greater temperature change for a specified forcing and (ii) the smaller CO_2_ change required to yield a given forcing from the lesser CO_2_ level of the higher sensitivity case. The average CO_2_ amount in the Early Pliocene is about 300 ppm for the Russell sensitivity, but could reach a few tens of ppm higher if the true sensitivity is closer to two-thirds of the Russell sensitivity.

### (b) Comparison with van de Wal *et al.* model

van de Wal *et al.* [123] used the same Zachos *et al.* [4] *δ*^18^O data to drive an inverse model calculation, including an ice sheet model to separate ice volume and temperature, thus inferring CO_2_ over the past 20 Myr. They find an MMCO CO_2_ approximately 450 ppm, which falls between the Russell and two-thirds Russell sensitivities (figure 9). The van de Wal *et al.* [123] model has a 30^°^C change in Northern Hemisphere temperature (their model is hemispheric) between the MMCO and average Pleistocene conditions driven by a CO_2_ decline from approximately 450 ppm to approximately 250 ppm, which is a forcing of approximately 3.5 W m^−2^. Thus, the implied (Northern Hemisphere) Earth system sensitivity is an implausible approximately 35^°^C for a 4 W m^−2^ CO_2_ forcing. The large temperature change may be required to produce substantial sea-level change in their ice sheet model, which we suggested above is unrealistically unresponsive to climate change. However, they assign most of the temperature change to slow feedbacks, thus inferring a fast-feedback sensitivity of only about 3^°^C per CO_2_ doubling.

### (c) Inferences from the Palaeocene–Eocene Thermal Maximum and Early Cenozoic climate

**Figure 10.**
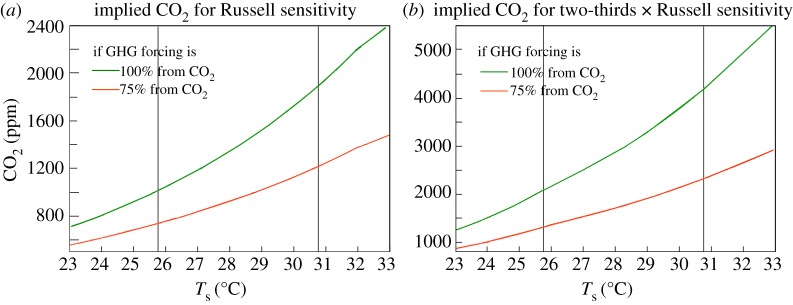
Atmospheric CO_2_ amount (*y*-axis) required to yield a given global temperature (*x*-axis) at the time of the PETM for (*a*) the Russell climate sensitivity and (*b*) two-thirds of the Russell sensitivity. The CO_2_ increment required to yield a given PETM warming above the pre-PETM temperature (25.7^°^C) is obtained by subtracting the CO_2_ amount at the desired *T*_s_ from the CO_2_ at *T*_s_=25.7^°^C. The vertical line is for the case of 5^°^C PETM warming. The orange lines show the required CO_2_ if the CO_2_ increase is accompanied by a non-CO_2_ GHG feedback that provides 25% of the total GHG forcing.

Finally, we use the largest and best documented of the hyperthermals, the PETM, to test the reasonableness of the Russell state-dependent climate sensitivity. Global warming in the PETM is reasonably well defined at 5–6^°^C and the plausible range for carbon mass input is approximately 4000–7000 Pg C [14]. Given that the PETM carbon injection occurred over a period of a few millennia, carbon cycle models suggest that about one-third of the carbon would be airborne as CO_2_ following complete injection [21]. With a conversion factor of 1 ppm CO_2_∼2.12 Gt C, the 4000–7000 Gt C source thus yields approximately 630–1100 ppm CO_2_. We can use figure 10, obtained via the same calculations as described above, to see how much CO_2_ is required to yield a 5^°^C warming. The Russell sensitivity requires approximately 800 ppm CO_2_ for a 5^°^C warming, whereas two-thirds of the Russell sensitivity requires approximately 2100 ppm CO_2_. Given the uncertainty in the airborne fraction of CO_2_ and possible non-CO_2_ gases, we cannot rule out the two-thirds Russell sensitivity, but the full Russell sensitivity fits plausible PETM carbon sources much better, especially if the PETM warming is actually somewhat more than 5^°^C (see figure 10 for quantitative implications).

This analysis is for Earth system sensitivity with CO_2_ as the forcing, as is appropriate for the PETM because any carbon injected as CH_4_ would be rapidly oxidized to CO_2_. Feedbacks in the PETM do not include large ice sheets, but non-CO_2_ GHGs are an unmeasured feedback. If a warming climate increases the amount of N_2_O and CH_4_ in the air, the required carbon source for a given global warming is reduced, because the amount of carbon in airborne CH_4_ is negligible. Any non-CO_2_ GHG feedback increases the CO_2_-forced Earth system sensitivity, potentially by a large amount (figure 10). The CO_2_-forced Earth sensitivity is the most relevant climate sensitivity, not only for the PETM but for human-made forcings. Although present enhanced amounts of airborne CH_4_ and N_2_O are mostly a climate forcing, i.e. their increases above the pre-industrial level are mainly a consequence of human-made sources, they also include a GHG feedback. Climate sensitivity including this GHG feedback is the most relevant sensitivity for humanity as the CO_2_ forcing continues to grow.

If the EECO global temperature exceeded 28^°^C, as suggested by multi-proxy data taken at face value (see above), climate sensitivity implied by the EECO warmth and the PETM warming is close to the full Russell climate sensitivity (see electronic supplementary material, figures S7–S9). We conclude that the existing data favour a climate sensitivity of at least two-thirds of the Russell sensitivity, and probably closer to the full Russell sensitivity. That lower limit is just over 3^°^C for 2×CO_2_ for the range of climate states of immediate relevance to humanity (figure 7*b*).

## 7. Summary discussion

Covariation of climate, sea level and atmospheric CO_2_ through the Cenozoic era is a rich source of information that can advise us about the sensitivity of climate and ice sheets to forcings, including human-made forcings. Our approach is to estimate Cenozoic sea level and temperature from empirical data, with transparent assumptions and minimal modelling. Our data are available in the electronic supplementary material, allowing comparison with other data and model results.

### (a) Sea-level sensitivity

Hansen [49,50] argues that real ice sheets are more responsive to warming than in most ice sheet models, which suggests that large ice sheets are relatively stable. The model of Pollard & DeConto [124], for example, requires three to four times the pre-industrial CO_2_ amount to melt the Antarctic ice sheet. This stability is, in part, a result of hysteresis: as the Earth warms, the ice sheet size as a function of temperature does not return on the same path that it followed as temperature fell and the ice sheet grew. We do not question the reality of mechanisms that cause ice sheet hysteresis, but we suspect they are exaggerated in models. Thus, as an extreme alternative that can be compared with ice sheet models and real-world data, we assume that hysteresis effects are negligible in our approximation for sea level as a function of temperature.

Ice sheets in question are those on Greenland and Antarctica, ice sheets that could shrink with future warming. Despite the stability of those ice sheets in the Holocene, there is evidence that sea level was much more variable during the Eemian, when we estimate the peak global temperature was only +1.0^°^C warmer than in the first decade of the twenty-first century. Rohling *et al.* [52] estimate an average rate of Eemian sea-level change of 1.4 m per century, and several studies noted above suggest that the Eemian sea level reached heights of +4–6 m or more relative to today.

The MMCO provides one test of hysteresis. Our sea-level approximation (figure 2) suggests that the Antarctic ice sheet nearly disappeared at that time. John *et al.* [125] provide support for that interpretation, as well as evidence of numerous rises and falls of sea level by 20–30 m during the Miocene. These variations are even larger than those we find (figure 2), but the resolution of the *δ*^18^O data we use is not adequate to provide the full amplitude of variations during that period (electronic supplementary material, figure S1).

The Mid-Pliocene is a more important test of ice sheet variability. We find sea-level fluctuations of at least 20–40 m, much greater than in ice sheet models (figure 2), with global temperature variations of only a few degrees. Independent analyses designed to separate ice volume and temperature change, such as Dwyer & Chandler [64], find sea-level maxima and variability comparable to our estimates. Altogether, the empirical data support a high sensitivity of the sea level to global temperature change, and they provide strong evidence against the seeming lethargy and large hysteresis effects that occur in at least some ice sheet models.

### (b) Fast-feedback climate sensitivity

Estimates of climate sensitivity cover a wide range that has existed for decades [1,48,99]. That range measures our ignorance; it does not mean that climate response from a specified state is stochastic with such inherent uncertainty. God (Nature) plays dice, but not for such large amounts. Indeed, one implication of the tight fit of calculated and measured temperature change of the past 800 000 years (figure 6) is that there is a single well-defined, but unknown, fast-feedback global climate sensitivity for that range of climate, despite large regional climate variations and ocean dynamical effects [31].

Improved empirical data can define climate sensitivity much more precisely, provided that climate-induced aerosol changes are included in the category of fast feedbacks (human-made aerosol changes are a climate forcing). Empirical assessment of fast-feedback climate sensitivity is obtained by comparing two quasi-equilibrium climate states for which boundary condition climate forcings (which may be slow feedbacks) are known. Aerosol changes between those climate states are appropriately included as a fast feedback, not only because aerosols respond rapidly to changing climate but also because there are multiple aerosol compositions, they have complex radiative properties and they affect clouds in several ways, thus making accurate knowledge of their glacial–interglacial changes inaccessible.

The temporal variation of the GHG plus surface albedo climate forcing closely mimics the temporal variation of either the deep ocean temperature (figure 6) or Antarctic temperature [5,31] for the entire 800 000 years of polar ice core data. However, the temperature change must be converted to the global mean to allow inference of climate sensitivity. The required scale factor is commonly extracted from an estimated LGM–Holocene global temperature change, which, however, is very uncertain, with estimates ranging from approximately 3^°^C to approximately 6^°^C. Thus, for example, the climate sensitivity (1.7–2.6^°^C for 2×CO_2_) estimated by Schmittner *et al.* [94] is due largely to their assumed approximately 3^°^C cooling in the LGM, and in lesser part to the fact that they defined some aerosol changes (dust) to be a climate forcing.

Climate sensitivity extracted from Pleistocene climate change is thus inherently partly subjective as it depends on how much weight is given to mutually inconsistent estimates of glacial-to-interglacial global temperature change. Our initial assessment is a fast-feedback sensitivity of 3±1^°^C for 2×CO_2_, corresponding to an LGM cooling of 4.5^°^C, similar to the 2.2–4.8^°^C estimate of PALAEOSENS [99]. This sensitivity is higher than estimated by Schmittner *et al.* [94], partly because they included natural aerosol changes as a forcing. In addition, we note that their proxies for LGM sea surface cooling exclude planktic foraminifera data, which suggest larger cooling [126], and, as noted by Schneider von Deimling *et al.* [95], regions that are not sampled tend to be ones where the largest cooling is expected. It should be possible to gain consensus on a narrower range for climate sensitivity via a community project for the LGM analogous to PRISM Pliocene data reconstruction [97,98] and PlioMIP model intercomparisons [67,68].

However, we suggest that an even more fruitful approach would be a focused effort to define the glacial-to-interglacial climate change of the Eemian period (MIS-5e). The Eemian avoids the possibility of significant human-made effects, which may be a factor in the Holocene. Ruddiman [127] suggests that deforestation and agricultural activities affected CO_2_ and CH_4_ in the Holocene, and Hansen *et al.* [91] argue that human-made aerosols were probably important. Given the level of Eemian warmth, approximately +1.8^°^C relative to 1880–1920, with a climate forcing similar to that for LGM–Holocene (figure 5), we conclude that this relatively clean empirical assessment yields a fast-feedback climate sensitivity in the upper part of the range suggested by the LGM–Holocene climate change, i.e. a sensitivity of 3–4^°^C for 2×CO_2_. Detailed study is especially warranted because Eemian warmth is anticipated to recur in the near term.

### (c) Earth system sensitivity

We have shown that global temperature change over the Cenozoic era is consistent with CO_2_ change being the climate forcing that drove the long-term climate change. Proxy CO_2_ measurements are so variable and uncertain that we only rely on the conclusion that the CO_2_ amount was of the order of 1000 ppm during peak Early Eocene warmth. That conclusion, in conjunction with a climate model incorporating only the most fundamental processes, constrains average fast-feedback climate sensitivity to be in the upper part of the sensitivity range that is normally quoted [1,48,99], i.e. the sensitivity is greater than 3^°^C for 2×CO_2_. Strictly this Cenozoic evaluation refers to the average fast-feedback sensitivity for the range of climates from ice ages to peak Cenozoic warmth and to the situation at the time of the PETM. However, it would be difficult to achieve that high average sensitivity if the current fast-feedback sensitivity were not at least in the upper half of the range of 3±1^°^C for 2×CO_2_.

This climate sensitivity evaluation has implications for the atmospheric CO_2_ amount throughout the Cenozoic era, which can be checked as improved proxy CO_2_ measurements become available. The CO_2_ amount was only approximately 450–500 ppm 34 Myr BP when large-scale glaciation first occurred on Antarctica. Perhaps more important, the amount of CO_2_ required to melt most of Antarctica in the MMCO was only approximately 450–500 ppm, conceivably only about 400 ppm. These CO_2_ amounts are smaller than suggested by ice sheet/climate models, providing further indication that the ice sheet models are excessively lethargic, i.e. resistant to climate change. The CO_2_ amount in the earliest Pliocene, averaged over astronomical cycles, was apparently only about 300 ppm, and decreased further during the Pliocene.

### (d) Runaway greenhouse

Our climate simulations, using a simplified three-dimensional climate model to solve the fundamental equations for conservation of water, atmospheric mass, energy, momentum and the ideal gas law, but stripped to basic radiative, convective and dynamical processes, finds upturns in climate sensitivity at the same forcings as found with a more complex global climate model [66]. At forcings beyond these points the complex model ‘crashed’, as have other climate models (discussed by Lunt *et al.* [83]). The upturn at the 10–20 W m^−2^ negative forcing has a simple physical explanation: it is the snowball Earth instability. Model crashes for large positive forcings are sometimes described as a runaway greenhouse, but they probably are caused by one of the many parametrizations in complex global models going outside its range of validity, not by a runaway greenhouse effect.

The runaway greenhouse effect has several meanings ranging from, at the low end, global warming sufficient to induce out-of-control amplifying feedbacks, such as ice sheet disintegration and melting of methane hydrates, to, at the high end, a Venus-like hothouse with crustal carbon baked into the atmosphere and a surface temperature of several hundred degrees, a climate state from which there is no escape. Between these extremes is the moist greenhouse, which occurs if the climate forcing is large enough to make H_2_O a major atmospheric constituent [106]. In principle, an extreme moist greenhouse might cause an instability with water vapour preventing radiation to space of all absorbed solar energy, resulting in very high surface temperature and evaporation of the ocean [105]. However, the availability of non-radiative means for vertical transport of energy, including small-scale convection and large-scale atmospheric motions, must be accounted for, as is done in our atmospheric general circulation model. Our simulations indicate that no plausible human-made GHG forcing can cause an instability and runaway greenhouse effect as defined by Ingersoll [105], in agreement with the theoretical analyses of Goldblatt & Watson [128].

On the other hand, conceivable levels of human-made climate forcing could yield the low-end runaway greenhouse. A forcing of 12–16 W m^−2^, which would require CO_2_ to increase by a factor of 8–16 times, if the forcing were due only to CO_2_ change, would raise the global mean temperature by 16–24^°^C with much larger polar warming. Surely that would melt all the ice on the planet, and probably thaw methane hydrates and scorch carbon from global peat deposits and tropical forests. This forcing would not produce the extreme Venus-like baked-crust greenhouse state, which cannot be reached until the ocean is lost to space. A warming of 16–24^°^C produces a moderately moist greenhouse, with water vapour increasing to about 1% of the atmosphere's mass, thus increasing the rate of hydrogen escape to space. However, if the forcing is by fossil fuel CO_2_, the weathering process would remove the excess atmospheric CO_2_ on a time scale of 10^4^–10^5^ years, well before the ocean is significantly depleted. Baked-crust hothouse conditions on the Earth require a large long-term forcing that is unlikely to occur until the sun brightens by a few tens of per cent, which will take a few billion years [121].

### (e) Global habitability

Burning all fossil fuels would produce a different, practically uninhabitable, planet. Let us first consider a 12 W m^−2^ greenhouse forcing, which we simulated with 8×CO_2_. If non-CO_2_ GHGs such as N_2_O and CH_4_ increase with global warming at the same rate as in the palaeoclimate record and atmospheric chemistry simulations [122], these other gases provide approximately 25% of the greenhouse forcing. The remaining 9 W m^−2^ forcing requires approximately 4.8×CO_2_, corresponding to fossil fuel emissions as much as approximately 10,000 Gt C for a conservative assumption of a CO_2_ airborne fraction averaging one-third over the 1000 years following a peak emission [21,129].

Our calculated global warming in this case is 16^°^C, with warming at the poles approximately 30^°^C. Calculated warming over land areas averages approximately 20^°^C. Such temperatures would eliminate grain production in almost all agricultural regions in the world [130]. Increased stratospheric water vapour would diminish the stratospheric ozone layer [131].

More ominously, global warming of that magnitude would make most of the planet uninhabitable by humans [132,133]. The human body generates about 100 W of metabolic heat that must be carried away to maintain a core body temperature near 37^°^C, which implies that sustained wet bulb temperatures above 35^°^C can result in lethal hyperthermia [132,134]. Today, the summer temperature varies widely over the Earth's surface, but wet bulb temperature is more narrowly confined by the effect of humidity, with the most common value of approximately 26–27^°^C and the highest approximately of 31^°^C. A warming of 10–12^°^C would put most of today's world population in regions with wet a bulb temperature above 35^°^C [132]. Given the 20^°^C warming we find with 4.8×CO_2_, it is clear that such a climate forcing would produce intolerable climatic conditions even if the true climate sensitivity is significantly less than the Russell sensitivity, or, if the Russell sensitivity is accurate, the CO_2_ amount required to produce intolerable conditions for humans is less than 4.8×CO_2_. Note also that increased heat stress due to warming of the past few decades is already enough to affect health and workplace productivity at low latitudes, where the impact falls most heavily on low- and middle-income countries [135].

The Earth was 10–12^°^C warmer than today in the Early Eocene and at the peak of the PETM (figure 4). How did mammals survive that warmth? Some mammals have higher internal temperatures than humans and there is evidence of evolution of surface-area-to-mass ratio to aid heat dissipation, for example transient dwarfing of mammals [136] and even soil fauna [137] during the PETM warming. However, human-made warming will occur in a few centuries, as opposed to several millennia in the PETM, thus providing little opportunity for evolutionary dwarfism to alleviate impacts of global warming. We conclude that the large climate change from burning all fossil fuels would threaten the biological health and survival of humanity, making policies that rely substantially on adaptation inadequate.

Let us now verify that our assumed fossil fuel climate forcing of 9 W m^−2^ is feasible. If we assume that fossil fuel emissions increase by 3% per year, typical of the past decade and of the entire period since 1950, cumulative fossil fuel emissions will reach 10 000 Gt C in 118 years. However, with such large rapidly growing emissions the assumed 33% CO_2_ airborne fraction is surely too small. The airborne fraction, observed to have been 55% since 1950 [1], should increase because of well-known nonlinearity in ocean chemistry and saturation of carbon sinks, implying that the airborne fraction probably will be closer to two-thirds rather than one-third, at least for a century or more. Thus, the fossil fuel source required to yield a 9 W m^−2^ forcing may be closer to 5000 Gt C, rather than 10 000 Gt C.

Are there sufficient fossil fuel reserves to yield 5000–10 000 Gt C? Recent updates of potential reserves [114], including unconventional fossil fuels (such as tar sands, tar shale and hydrofracking-derived shale gas) in addition to conventional oil, gas and coal, suggest that 5×CO_2_ (1400 ppm) is indeed feasible. For instance, using the emission factor for coal from IPCC [48], coal resources given by the Global Energy Assessment [114] amount to 7300–11 000 Gt C. Similarly, using emission factors from IPCC [48], total recoverable fossil energy reserves and resources estimated by GEA [114] are approximately 15 000 Gt C. This does not include large ‘additional occurrences’ listed in ch. 7 of GEA [114]. Thus, for a multi-centennial CO_2_ airborne fraction between one-third and two-thirds, as discussed above, there are more than enough available fossil fuels to cause a forcing of 9 W m^−2^ sustained for centuries.

Most of the remaining fossil fuel carbon is in coal and unconventional oil and gas. Thus, it seems, humanity stands at a fork in the road. As conventional oil and gas are depleted, will we move to carbon-free energy and efficiency—or to unconventional fossil fuels and coal? If fossil fuels were made to pay their costs to society, costs of pollution and climate change, carbon-free alternatives might supplant fossil fuels over a period of decades. However, if governments force the public to bear the external costs and even subsidize fossil fuels, carbon emissions are likely to continue to grow, with deleterious consequences for young people and future generations.

It seems implausible that humanity will not alter its energy course as consequences of burning all fossil fuels become clearer. Yet strong evidence about the dangers of human-made climate change have so far had little effect. Whether governments continue to be so foolhardy as to allow or encourage development of all fossil fuels may determine the fate of humanity.
